# 

**Published:** 1911-09

**Authors:** 


"Red Back"

PUBLISHED AT AUSTIN, TEXAS, BY

F. E. DANIEL, M. D., - - - - Editor and Proprietor.

PRINTED BY THE VON BOECKMANN-JONES CO.

**Subscription, $1.00 a Year, in Advance. - - - - Single Copies, 10 Cents**

Entered at the Postoffice at Austin, Texas, as second class mail matter.

